# Performance Improvement of Receivers Based on Ultra-Tight Integration in GNSS-Challenged Environments

**DOI:** 10.3390/s131216406

**Published:** 2013-12-02

**Authors:** Feng Qin, Xingqun Zhan, Gang Du

**Affiliations:** School of Aeronautics and Astronautics, Shanghai Jiao Tong University, No.800 Dongchuan Road, Shanghai 200240, China; E-Mails: qinfengbreeze@live.cn (F.Q.); kevindu@sjtu.edu.cn (G.D.)

**Keywords:** ultra-tight integration, GNSS-challenged environment, phase lock loop, dynamic stress noise, quality of the IMU

## Abstract

Ultra-tight integration was first proposed by Abbott in 2003 with the purpose of integrating a global navigation satellite system (GNSS) and an inertial navigation system (INS). This technology can improve the tracking performances of a receiver by reconfiguring the tracking loops in GNSS-challenged environments. In this paper, the models of all error sources known to date in the phase lock loops (PLLs) of a standard receiver and an ultra-tightly integrated GNSS/INS receiver are built, respectively. Based on these models, the tracking performances of the two receivers are compared to verify the improvement due to the ultra-tight integration. Meanwhile, the PLL error distributions of the two receivers are also depicted to analyze the error changes of the tracking loops. These results show that the tracking error is significantly reduced in the ultra-tightly integrated GNSS/INS receiver since the receiver's dynamics are estimated and compensated by an INS. Moreover, the mathematical relationship between the tracking performances of the ultra-tightly integrated GNSS/INS receiver and the quality of the selected inertial measurement unit (IMU) is derived from the error models and proved by the error comparisons of four ultra-tightly integrated GNSS/INS receivers aided by different grade IMUs.

## 1. Introduction

The global navigation satellite system (GNSS) can only work in those environments where the number of GNSS satellites in view is no less than four. This leads to inconveniences and difficulties for GNSS applications in high-dynamic or weak signal scenarios where it is not easy to acquire navigation satellites. Hence, integrated GNSS/INS navigation systems have been developed for these GNSS-challenged environments. Inertial navigation systems (INSs) are capable of high update rates, while GNSS has high navigation accuracy. By fusing them together, the strengths and weaknesses of GNSS receivers and INS uniquely complement each other. Generally, the three architectures of integrated navigation systems, listed in order of complexity, are loose integration, tight integration, and ultra-tight integration [1,2].

In the loose integration and tight integration, the GNSS needs stable and strong signals for navigation applications. However, these signals are difficult to receive in the environments where line of sight (LOS) to satellites is not readily available, e.g., urban areas, indoors and dense forest areas. Such environments either completely block the GNSS signals or attenuate them to a power level which is 10–30 dB lower than nominal signal power [3]. Therefore, the ultra-tight integration was developed for GNSS receivers to realize high-dynamic navigation under weak signal conditions, by aiding the tracking loops of receivers. The acquisition and tracking capabilities of receivers are improved by this ultra-tight integration method [4,5].

Gustafson at the Charles Stark Draper Laboratory proposed in 2000 an ultra-tightly integrated navigator with extended range code tracking [6]. This led many researchers to pay attention to the ultra-tight integration since it has better tracking and navigation performance than the standard receiver and the tight integration [7,8]. Nowadays, some successes have been achieved in the various investigations on ultra-tight integration, such as the demonstration of the anti-interference capacity [9,10], the design and implementation of the dual-mode GNSS/INS ultra-tight integration [11] and the (Micro Electro Mechanical System) MEMS ultra-tight integration [12]. Nevertheless, most previous research about ultra-tight integration was still mainly on the architecture and filter design of the ultra-tightly integrated GNSS/INS navigation system [13–15]. The performance comparisons of the standard receiver and the ultra-tightly integrated GNSS/INS receiver were usually achieved by some experimental analyses [16]. No research used models of tracking loop errors to analyze the performance improvements brought by the ultra-tight integration, although the error models can demonstrate the essence of any performance improvements. Compared to the previous research, this paper presents the sources and compositions of tracking loop errors in both the standard receiver and the ultra-tightly integrated GNSS/INS receiver, and establishes the mathematical formulas of every error as well. Based on the tracking loop error analysis and comparisons of the two receivers, the advantages of the ultra-tightly integrated GNSS/INS receiver are starkly evident, especially in the high dynamics scenario. Moreover, the error distributions illustrate the proportions of the major noise sources in the two receivers and the noise reduction brought about by the ultra-tight integration.

In the ultra-tightly integrated GNSS/INS receiver, the level of the performance improvement is impacted by the quality of the inertial measurement unit (IMU) used. Some researchers have compared the tracking and navigation performances of ultra-tight integrations with different grade IMUs by simulation experiments [7,15], but there has been no research to derive the mathematical relationship between the level of the performance improvement and the IMU quality. Hence, the mathematical relationship between the tracking performances of the ultra-tightly integrated GNSS/INS receiver and the quality of the selected IMU is built in this paper to make up for this insufficiency. To verify this relationship, some simulations are performed to compare the loop performances of four ultra-tightly integrated GNSS/INS receivers aided by different grade IMUs. This investigation is very valuable for the selection of the IMU in an ultra-tightly integrated GNSS/INS receiver.

There are two loops in receivers: delay lock loop (DLL) and phase lock loop (PLL). Compared to the DLL, the PLL is more sensitive to dynamic stress and it loses lock much easier since the carrier wavelength is much shorter than the code chip length. Therefore, the tracking performances of the PLL get more attention than that of the DLL. In this paper, PLL loop noises are analyzed to evaluate the improvement of the tracking performances.

## 2. Ultra-Tightly Integrated GNSS/INS Architecture

In the standard receiver, the received signals are tracked by scalar tracking loops. The receiver's dynamics cannot be compensated in tracking processes and tracking loops easily lose lock in weak signal environments. Hence, the ultra-tightly integrated GNSS/INS receiver which can withstand signal interferences and achieve robust signal acquisitions and trackings is proposed.

The primary advantage of the ultra-tight integration method is the inherent robustness in the presence of intentional jamming or unintentional interference. A second advantage is that this method offers improved tracking and more accurate navigation solutions. Consequently, the ultra-tightly integrated GNSS/INS receiver does not easily lose lock on the satellite signals because the ultra-tight method continuously correlates received and replica signals over the entire integration Kalman cycle for all satellites in view [17].

**Figure 1.**
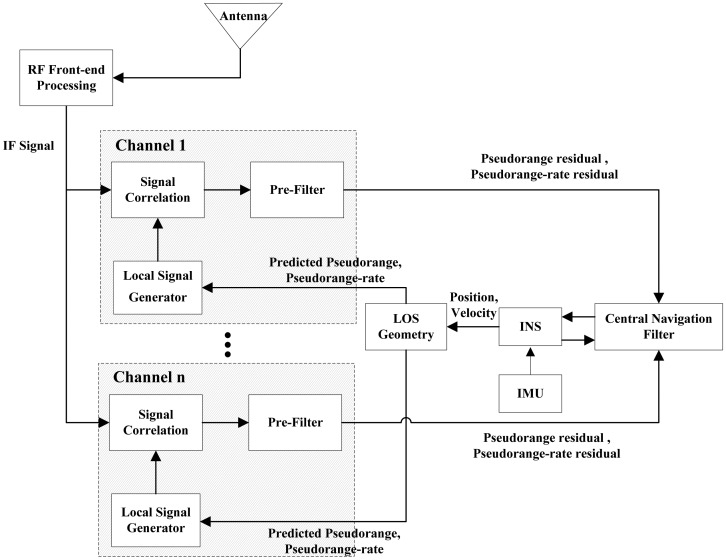
The architecture of the vector tracking-based ultra-tightly integrated GNSS/INS receiver.

**Figure 2.**
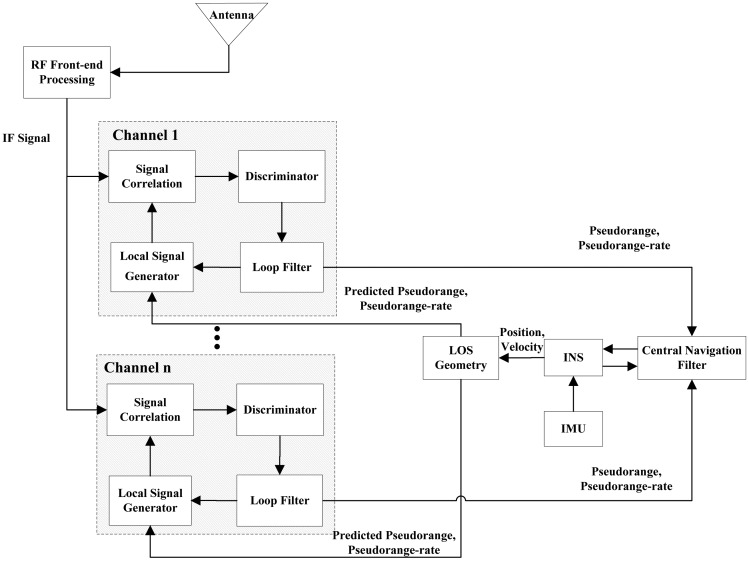
The architecture of the scalar tracking based ultra-tightly integrated GNSS/INS receiver.

There are two types of the ultra-tightly integrated GNSS/INS receiver. One is the vector tracking based ultra-tightly integrated GNSS/INS receiver, the other is the scalar tracking based ultra-tightly integrated GNSS/INS receiver. Figure 1 shows the architecture of the vector tracking-based ultra-tightly integrated GNSS/INS receiver, whereas Figure 2 shows the architecture of the scalar tracking-based ultra-tightly integrated GNSS/INS receiver.

In the vector tracking-based ultra-tightly integrated receiver, all tracking loops are coupled by a navigation filter. Each tracking loop includes six correlators, a pre-filter, a navigation filter, an aided parameter estimator and a local replica signal generator. The replica signals from all loops firstly correlate with received signals processed by a radio frequency (RF) front end. The in-phase (I) and quadra-phase (Q) outputs obtained from the correlators are used as the measurements of the pre-filters to estimate pseudorange residuals and pseudorange rate residuals. Then, these pseudorange and pseudorange rate residuals of all visible satellites are provided to the central navigation filter as the measurements needed to correct the position and velocity computed from an INS. Finally, the pseudoranges and pseudorange rates predicted from the corrected position and velocity by the LOS geometry algorithm are fed back to the local signal generators to adjust local replica signals [18,19].

Compared to the vector tracking loops, the tracking loops in the scalar tracking-based ultra-tightly integrated GNSS/INS receiver are independent each other. In this receiver, the INS aiding is added into the traditional scalar loops to estimate and compensated the vehicle's dynamics with respect to the satellites. The pseudorange and pseudorange-rate outputs obtained from the loop filters are provided to the central navigation filter as the measurements to correct the position and velocity computed from an INS. Then, the corrected position and velocity are further used to predict the pseudoranges and pseudorange rates for adjusting local replica signals.

The position and velocity outputs in the ultra-tight integration are obtained from the central navigation filter instead of the traditional navigation solution used in the standard receiver. The central navigation filter can estimate the receiver antenna's position and velocity, even though the number of the satellite measurements is less than four. Hence, the ultra-tightly integrated GNSS/INS receiver can achieve navigation in the environments where the number of GNSS satellites in view is less than four.

On the other hand, the GNSS-challenged environment where the number of visible satellites is less than four is caused by the high dynamics between the vehicle and the satellites and the low carrier to noise ratio density (C/N0) of the GNSS signal. In the ultra-tight integration, the vehicle's dynamics with the respect to the satellites are compensated by the INS aiding, and the lowest C/N0 accepted by the GNSS signal acquisition and tracking is reduced. Hence, some satellites which are considered not visible in the standard receiver can be acquired and tracked in the ultra-tightly integrated GNSS/INS receiver. The number of the GNSS satellites in view can increase in the ultra-tightly integrated GNSS/INS receiver due to the INS aid. In the following sections, the advantages of the ultra-tight integration in GNSS-challenged environments are analyzed in detail.

## 3. Tracking Loops Error Characterization

The precision of the receiver observations is affected by a set of factors. The most important one that limits the accuracy of a GNSS receiver is the tracking loop noise, including dynamic stress noise, thermal noise, Allan deviation phase noise and vibration-induced phase noise. As stated in [20], the thermal noise and dynamic stress noise mainly affect the correlations between received signals and replica signals, whereas the Allan deviation phase noise and vibration-induced phase noise have an effect on numerically controlled oscillators (NCOs). Since the noise sources of the DLL and PLL are identical and the PLL is more sensitive to noises, the performance analysis of the tracking loop focuses on the PLL in this paper.

### 3.1. Thermal Noise

The thermal noise in the PLL is related with the induced noise of electronic parts that compose a receiver. It is determined by the PLL bandwidth, carrier to noise ratio density, predetection integration time and carrier wavelength. The 1-sigma thermal noise error can be expressed as follows [21,22]:

(1) $$\begin{array}{lll} \sigma_{T} & =\frac{360}{2\pi }\sqrt{\frac{B_{\text{PLL}}}{c/n_{0}}\left(1+\frac{1}{2\cdot T_{\text{PLL}}\cdot c/n_{0}}\right)} & \left({}^{\circ}\right)\\ & =\frac{\lambda_{c}}{2\pi }\sqrt{\frac{B_{\text{PLL}}}{c/n_{0}}\left(1+\frac{1}{2\cdot T_{\text{PLL}}\cdot c/n_{0}}\right)} & \left(m\right) \end{array}$$

where *λ_c_* is the carrier wavelength (m). *B_PLL_* is the PLL bandwidth (Hz). *T_PLL_* is the predetection integration time (s). *c* / *n*_0_ is the carrier to noise power expressed as a ratio 10^(^*^C^*^/^*^N^*^0)/10^ (C/N0 expressed in [dB-Hz]).

### 3.2. Dynamic Stress Error

The dynamic stress error is associated to the motions that the receiver antenna suffers. It is inevitable in the PLL tracking and plays a major role in the tracking performance. The purpose of the ultra-tight integration is just to reduce the dynamic stress error by estimating and compensating the receiver antenna's LOS dynamics from the INS. The dynamic stress error (1-sigma) depends on loop order and described as follows [23]:

(2) $$\begin{array}{lll} \theta_{e}=\frac{1}{3}\cdot \frac{d^{k}R/dt^{k}}{\omega_{\text{PLL}}^{k}} & =\frac{1}{3}\cdot K_{k}\frac{d^{k}R/dt^{k}}{{\left(B_{\text{PLL}}\right)}^{k}} & \left(\text{m}\right)\\ & =\frac{1}{3}\cdot K_{k}\frac{d^{k}R/dt^{k}}{{\left(B_{\text{PLL}}\right)}^{k}}\cdot \frac{360\cdot L_{c}}{c} & \left({}^{\circ}\right) \end{array}$$

where *d^k^R*/*dt^k^* is the relative dynamics between the satellite and the GNSS receiver antenna in its LOS (m/s^k^). *B_PLL_* is the PLL bandwidth (Hz). *L_c_* is the carrier frequency (Hz). *c* is the free space speed of electromagnetic propagation (m/s). *K_k_* is the proportion coefficient. *k*=2, *K*_2_=0.2809 for a second-order PLL, *k*=3, *K*_3_=0.4828 for a third-order PLL.

### 3.3. Allan Deviation Phase Noise

The Allan deviation phase noise is caused by the drift of the receiver oscillator, which is determined by the oscillator's material and craft. The phase noise induced by the frequency drift can be expressed as follows [20]:

(3) $$\sigma_{A}^{2}=\frac{1}{2\pi }\int_{0}^{\infty }S_{\phi }\left(\omega \right){\left|1-H\left(\omega \right)\right|}^{2}d\omega$$

where *S_φ_* (*ω*) is the single-sided oscillator phase noise spectrum density. ${\left|1-H\left(\omega \right)\right|}^{2}=\frac{\omega^{2n}}{\omega_{L}^{2n}+\omega^{2n}}$ is the parameter which depends on loop order. In the equation, *ω_L_* can be computed by using *ω_L_*,_1_ = 4*B_PLL_*, *ω_L_*,_2_ = 1.885*B_PLL_*, *ω_L_*,_3_ =1.2*B_PLL_* for first-order, second-order, and third-order PLLs, respectively.

In Equation (3)*S_φ_* (*ω*) can be described as follows:

(4) $$S_{\phi }\left(\omega \right)={\left(2\pi L_{c}\right)}^{2}\cdot \frac{S_{y}\left(\omega \right)}{\omega^{2}}$$

where *L_c_* is the carrier frequency. *S_y_* (*ω*) is the clock's power spectrum, which can be expressed as follows. The flicker phase and white phase noise is omitted due to their weak contribution on the oscillator error:

(5) $$S_{y}\left(\omega \right)=\frac{2\pi^{2}h_{-2}}{\omega^{2}}+\frac{\pi h_{-1}}{\omega }+\frac{h_{0}}{2}$$

Combining Equation (3), Equation (4) and Equation (5), the Allan deviation phase noise is derived for a second-order PLL:

(6) $$\begin{array}{ccc} \sigma_{A}^{2} & =2\pi L_{c}^{2}\int_{0}^{\infty }\left[\frac{2\pi^{2}h_{-2}}{\omega^{4}}+\frac{\pi h_{-1}}{\omega^{3}}+\frac{h_{0}}{2\omega^{2}}\right]\frac{\omega^{4}}{\omega_{L}^{4}+\omega^{4}}d\omega & \\ & =2\pi^{2}L_{c}^{2}\left[\frac{\pi^{2}h_{-2}}{\sqrt{2}\omega_{L}^{3}}+\frac{\pi h_{-1}}{4\omega_{L}^{2}}+\frac{h_{0}}{4\sqrt{2}\omega_{L}}\right] & \left(\text{rad}\right)\\ & =2\times 180^{2}\cdot L_{c}^{2}\left[\frac{\pi^{2}h_{-2}}{\sqrt{2}\omega_{L}^{3}}+\frac{\pi h_{-1}}{4\omega_{L}^{2}}+\frac{h_{0}}{4\sqrt{2}\omega_{L}}\right] & \left({}^{\circ}\right) \end{array}$$

In this equation, the clock parameters *h*_−2_, *h*_−1_, and *h*_0_ listed in Table 1 represent the frequency stability of a certain oscillator.

### 3.4. Vibration-Induced Phase Noise

The vibration-induced phase noise is associated to the jitter in the receiver clock because of environmental vibrations. The model of the phase noise induced by environmental vibrations is similar to the Allan deviation phase noise. The model is written as follows:

(7) $$\sigma_{\phi }^{2}=\frac{1}{2\pi }\int_{0}^{\infty }G_{\phi }\left(\omega \right){\left|1-H\left(\omega \right)\right|}^{2}d\omega$$

In Equation (7) *G_φ_* (*ω*) is the single-sideband oscillator phase noise spectrum density and expressed as Equation (8) for the vibration-induced phase noise [20,24]:

(8) $$G_{\phi }\left(\omega \right)={\left(2\pi L_{c}\right)}^{2}\cdot k_{g}{\left(\omega \right)}^{2}\cdot \frac{G_{g}\left(\omega \right)}{\omega^{2}}$$

where *k_g_* (*ω*) is the oscillator's g-sensitivity in parts-per-g (parts/g). *G_g_* (*ω*) is the single-sided power spectrum density of vibrations (g^2^/Hz).

Substituting *G_φ_* (*ω*) from Equation (8) into Equation (7), the vibration-induced phase noise is derived for second-order PLL:

(9) $$\begin{array}{lll} \sigma_{\phi }^{2} & =2\pi L_{c}^{2}{k_{g}}^{2}G_{g}\int_{\omega 1}^{\omega 2}\frac{\omega^{2}}{\omega_{L}^{4}+\omega^{4}}d\omega & \\ & =\frac{\pi L_{c}^{2}k_{g}^{2}G_{g}}{\sqrt{2}\omega_{L}}\{\arctan\left(\frac{\omega_{2}\sqrt{2}}{\omega_{L}}+1\right)+\arctan\left(\frac{\omega_{2}\sqrt{2}}{\omega_{L}}-1\right) & \\ & \;-\arctan\left(\frac{\omega_{1}\sqrt{2}}{\omega_{L}}+1\right)-\arctan\left(\frac{\omega_{1}\sqrt{2}}{\omega_{L}}-1\right)+\frac{1}{2}\ln & \\ & \;\times \left[\frac{\left(\omega_{1}^{2}+\omega_{L}\omega_{1}\sqrt{2}+\omega_{L}^{2}\right)\left(\omega_{2}^{2}-\omega_{L}\omega_{2}\sqrt{2}+\omega_{L}^{2}\right)}{\left(\omega_{1}^{2}-\omega_{L}\omega_{1}\sqrt{2}+\omega_{L}^{2}\right)\left(\omega_{2}^{2}+\omega_{L}\omega_{1}\sqrt{2}+\omega_{L}^{2}\right)}\right]\} & \left(\text{rad}\right) \end{array}$$

### 3.5. Tracking Threshold of the PLL

The tracking threshold of the PLL is the maximum error accepted by the receiver to keep the PLL locked. It is usually obtained from multiple tracking experiments. However, these experiments are complicated and need to be repeated constantly in order to calculate the optimal tracking threshold. Hence, an empirical value (1-sigma) is gained from [23,25,26] for the analysis in this paper. Since the tracking loop error consists of the dynamic stress noise, the thermal noise, the Allan deviation phase noise and the vibration-induced phase noise, the criterion to keep the loop locked is described as follows:

(10) $$\sigma_{\text{PLL}}=\sigma_{T}+\theta_{e}+\sigma_{A}+\sigma_{\phi }\leq 15{}^{\circ}$$

In this equation, 15° is the 1-sigma empirical threshold.

## 4. Tracking Loops Error in the Ultra-Tightly Integrated GNSS/INS Receiver

In the ultra-tightly integrated GNSS/INS receiver, the dynamic stress noise is reduced by using an additional IMU to measure and compensate the dynamics experienced by the receiver antenna. After the dynamics are compensated, the dynamic stress noise for second-order PLL is mainly affected by the acceleration measurement error caused by the IMU errors, not by the acceleration of the motions that the receiver antenna experiences in LOS. Therefore, the dynamic stress noise (1-sigma) for second-order PLL in the ultra-tightly integrated GNSS/INS receiver is expressed according to [27]:

(11) $$\theta_{UT}=\frac{1}{3}\cdot K_{2}\frac{\delta \bar{a}}{{\left(B_{\text{PLL}}\right)}^{2}}\cdot \frac{360\cdot L_{c}}{c}$$

In the equation, *δā* is the measurement error vector of the acceleration in LOS.

Meanwhile, the acceleration error vector caused by the IMU can be modeled by:

(12) $$\delta {\bar{f}}_{\text{IMU}}=\bar{\nabla }+\bar{g}\cdot \bar{\alpha }\left(0\right)+\bar{g}\cdot \bar{ɛ}\cdot t_{d}$$

where ∇̄ is the accelerometer bias. *ε̄* is the gyro bias. *ḡ* is the acceleration vector of gravity. *ᾱ* (0) is the initial misalignment error. *t_d_* is the drift time if the IMU is unaided.

Since the acceleration of the pseudorange is measured by the IMU in LOS, the *δa* in Equation (11) is substituted by the *δ f_IMU_* in Equation (12). Therefore, the dynamic stress noise of the PLL in the ultra-tightly integrated GNSS/INS receiver is dependent on the IMU errors and can be expressed for second-order loop:

(13) $$\theta_{UT}=\frac{1}{3}\cdot K_{2}\frac{\delta {\bar{f}}_{\text{IMU}}}{{\left(B_{\text{PLL}}\right)}^{2}}\cdot \frac{360\cdot L_{c}}{c}$$

Similar to Equation (10), the tracking error of the PLL in the ultra-tightly integrated GNSS/INS receiver can be expressed:

(14) $$\sigma_{\text{PLL}}=\sigma_{T}+\theta_{UT}+\sigma_{A}+\sigma_{\phi }\leq 15{}^{\circ}$$

## 5. Tracking Performance Comparisons between Standard Receivers and Ultra-Tightly Integrated GNSS/INS Receivers

In the ultra-tightly integrated GNSS/INS receiver, the tracking performances can be improved as the dynamic stress noise is reduced. According to the models analyzed in this paper, the PLL performances in the standard receiver and the ultra-tightly integrated GNSS/INS receiver are compared to verify the improvement induced by the ultra-tight integration. In the comparisons, an IMU is selected to estimate the acceleration of the pseudorange.

**Figure 3.**
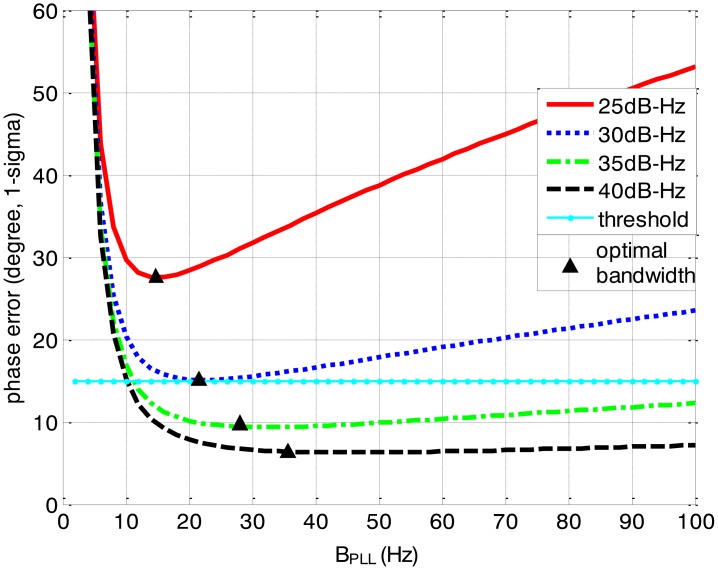
2nd loop phase error *vs*. B_PLL_ with different C/N_0_ in the standard receiver (0.5 g LOS acceleration).

**Figure 4.**
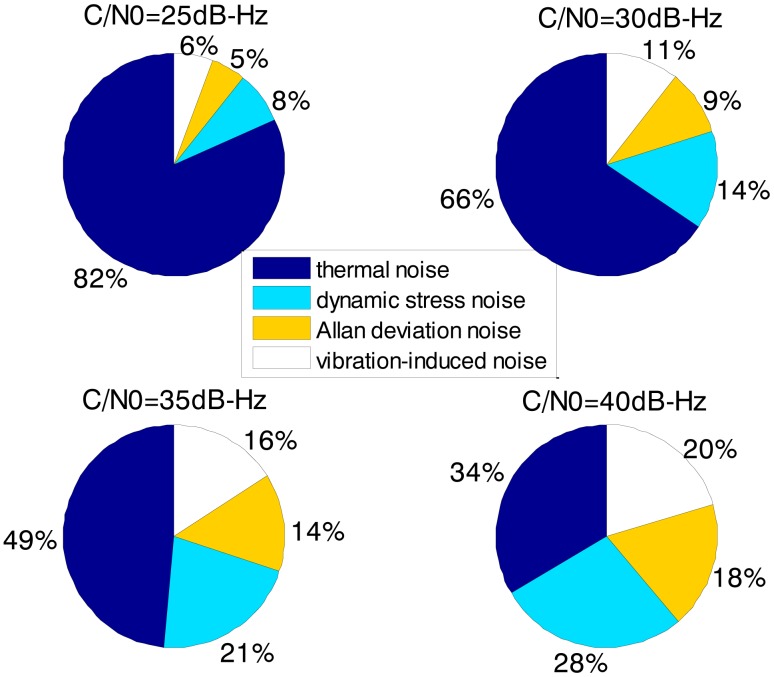
2nd loop error distributions with different C/N_0_ in the standard receiver (0.5 g LOS acceleration, 20 Hz PLL bandwidth).

**Figure 5.**
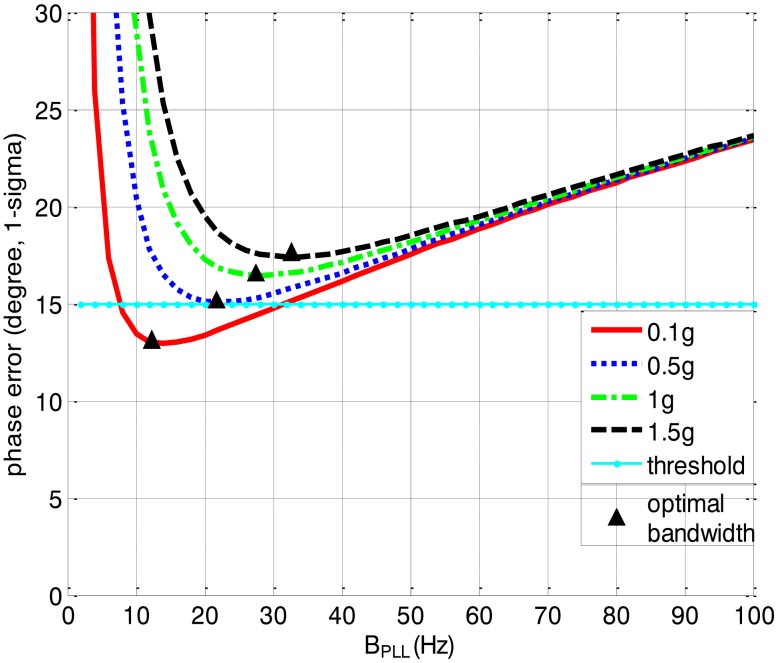
2nd loop phase error *vs*. B_PLL_ with different dynamics in the standard receiver (30 dB-Hz C/N0).

**Figure 6.**
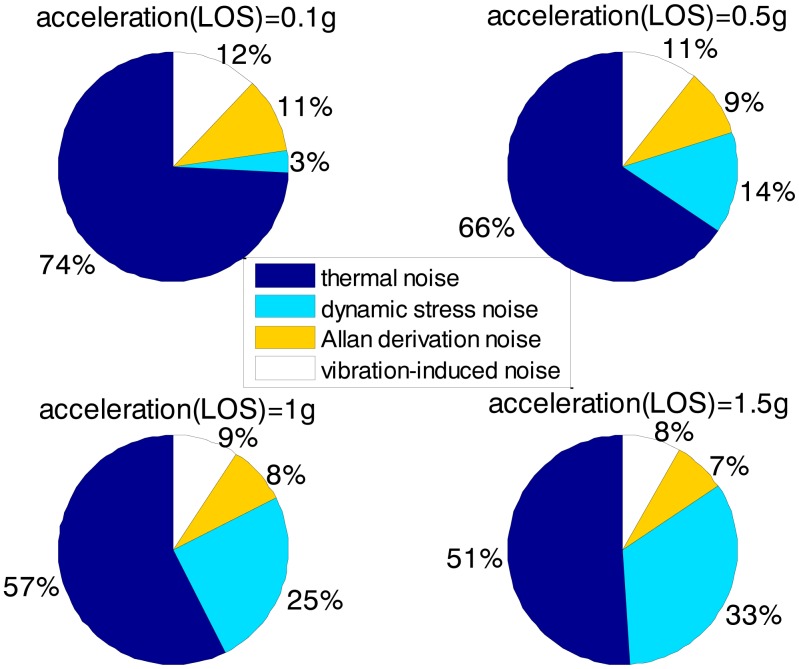
2nd loop error distributions with different dynamics in the standard receiver (30 dB-Hz C/N0, 20 Hz PLL bandwidth).

The gyro bias and accelerometer bias of the IMU are respectively 50°/h and 1 mg. The integration time length of the correlators is 1 ms. Moreover, a temperature compensated crystal oscillator (TCXO) is selected and the oscillator's g-sensitivity is 5 parts/g. Figures 3, 4, 5 and 6 depict the tracking performances in the standard receiver for second-order PLL. The tracking threshold which keeps the PLL locked is 15° in the simulations. The minimum loop error can be obtained when the loop bandwidth is optimal.

Figures 3 and 4 together illustrate the influence of C/N0 on the phase error of the PLL. Figure 3 shows the change of the overall phase error in different C/N0 scenarios, and then Figure 4 further analyzes which error source causes this change. As shown in Figure 3, when the LOS acceleration is assumed to be 0.5 g, the C/N0 of the GNSS signal for the unaided second-order PLL in the standard receiver should be no less than 30 dB-Hz to keep the loop locked. Hence, the GNSS signal whose C/N0 is less than 30 dB-Hz is defined as a challenge to the standard receiver in this paper. The higher is the C/N0, the less is the PLL phase error and the larger is the range of the loop bandwidth to keep the loop in lock. When the PLL bandwidth is assumed to be 20 Hz and the receiver antenna's acceleration in LOS is 0.5 g, the second-order PLL error distributions depicted in Figure 4 demonstrate that the thermal noise proportion decreases as the C/N0 increases. Since the other errors keep constant due to same PLL bandwidth and dynamic in different C/N0 scenarios, the decrease of the thermal noise proportion is contributed by the lessened thermal noise. Hence, we can further conclude that the reduction of the loop error in higher C/N0 scenarios is mainly caused by the reduction of the thermal noise. Similar to Figures 3 and 4, when the C/N0 is assumed to be 30 dB-Hz, Figure 5 illustrates that the LOS acceleration for the unaided second-order PLL should be no more than 0.5 g to keep the loop in lock. Hence, the receiver antenna's acceleration in LOS which is more than 0.5 g is defined as a challenge to the standard receiver in this paper. The loop error increases as the acceleration of the receiver antenna increases if the C/N0 and loop bandwidth are constant. These conclusions can be further proved by the change of the dynamic stress noise proportion with constant other noises in Figure 6. In a word, the thermal noise is mainly determined by the C/N0, whereas the dynamic stress noise is decided by the receiver antenna's dynamic in LOS.

**Figure 7.**
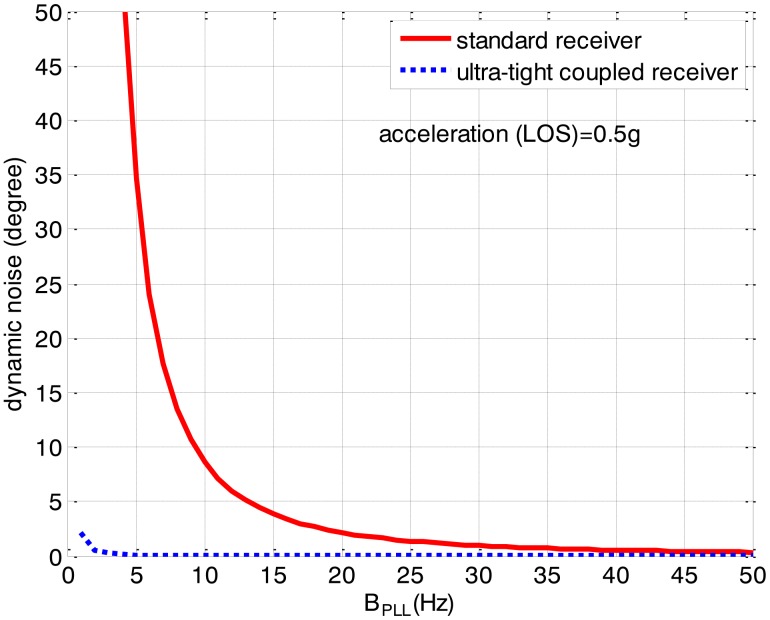
Dynamic stress noise comparisons between the standard receiver and the ultra-tightly integrated GNSS/INS receiver (0.5 g LOS acceleration).

To reduce the loop error and improve the tracking performances, the ultra-tight integration is designed to reduce the dynamic stress noise. Since the tracking loop is aided by an INS, the dynamic stress error in the ultra-tightly integrated GNSS/INS receiver is associated to the IMU errors and not related with the dynamics of the receiver antenna in LOS. Therefore, in the ultra-tightly integrated GNSS/INS receiver, the dynamic stress noise is evidently reduced and does not change in different dynamics. As is seen in Figure 7, the dynamic stress noise of the ultra-tightly integrated GNSS/INS receiver is obviously less than that of the standard receiver and its value is close to zero.

**Figure 8.**
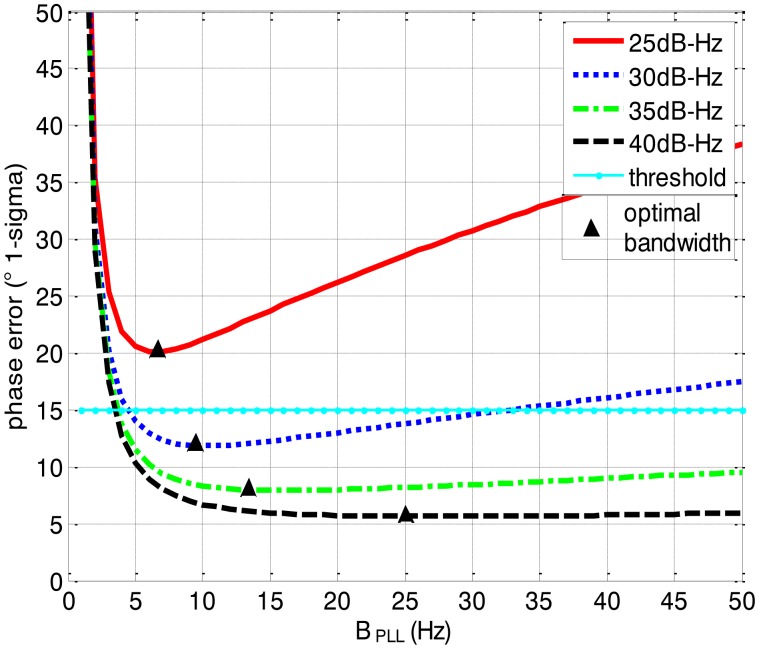
Loop phase error *vs*. B_PLL_ with different C/N_0_ in the ultra-tightly integrated GNSS/INS receiver.

**Figure 9.**
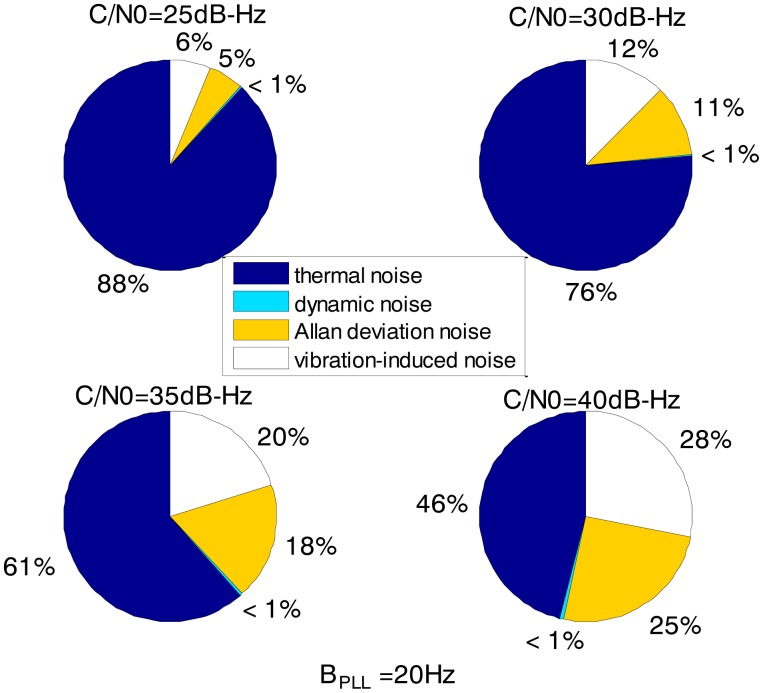
Loop error distributions with different C/N_0_ in the ultra-tightly integrated GNSS/INS receiver (20 Hz bandwidth).

**Figure 10.**
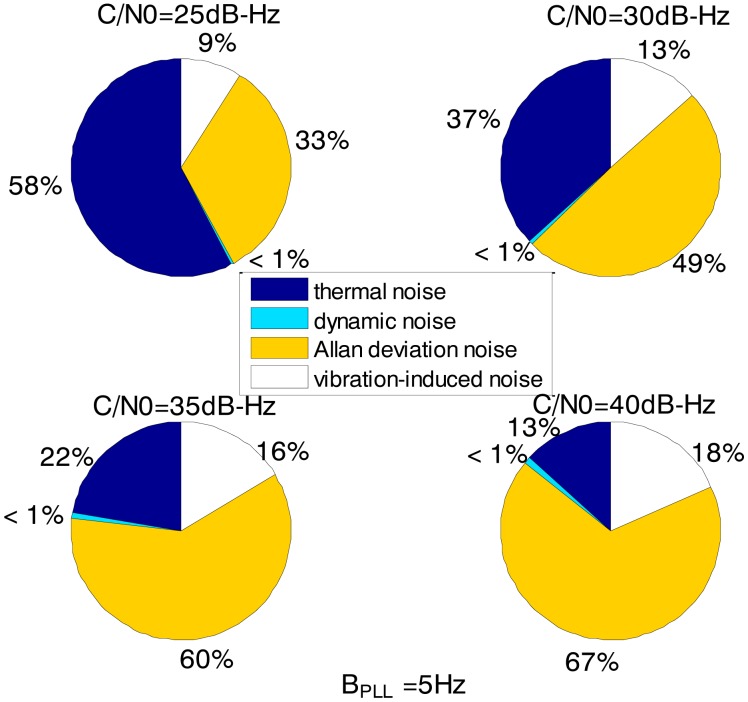
Loop error distributions with different C/N_0_ in the ultra-tightly integrated GNSS/INS receiver (5 Hz bandwidth).

Figures 8, 9 and 10 depict the tracking performances of the ultra-tightly integrated GNSS/INS receiver aided by the INS. The tracking threshold is as same as that of the standard receiver.

In Figure 8, it can be seen that the phase error of the PLL in the ultra-tightly integrated GNSS/INS receiver is less than that in the standard receiver with same C/N0. Due to the reduction of the phase error, the PLL in the ultra-tightly integrated GNSS/INS receiver is more stable and does not easily lose lock. Moreover, the phase error of the PLL in the ultra-tightly integrated GNSS/INS receiver is not related with the receiver antenna's dynamics, thus the results in Figure 8 have no constraint on the receiver antenna's dynamics. This receiver can perform well even in the high dynamic scenario.

The loop bandwidth of the PLL in the ultra-tightly integrated GNSS/INS receiver is assumed to be 20 Hz, which is the same as in the standard receiver. The error distributions in Figure 9 show that the dynamic stress noise proportion is less than 1% in the ultra-tightly integrated GNSS/INS receiver when the other noises are same as those in the standard receiver. Hence, we can conclude that the reduction of the loop error in the ultra-tightly integrated GNSS/INS receiver is brought by the compensation of the receiver antenna's dynamics. Since the phase error of the PLL is reduced, the ultra-tightly integrated GNSS/INS receiver can keep tracking in lower C/N0. It can acquire and track the weaker signal than the standard receiver.

Comparing Figure 8 with Figure 3, it can be derived that the ultra-tight integration can reduce the minimum PLL bandwidth to keep the loop in lock since the receiver antenna's dynamics is estimated and compensated. According to Equation (1), the thermal noise would decrease once the PLL bandwidth is reduced. Hence, not only the dynamic stress noise but also the thermal noise would decrease in the ultra-tightly integrated GNSS/INS receiver since the less PLL bandwidth can be selected. In this case, the Allan deviation noise and vibration-induced noise becomes the major error sources. It is consistent with the distributions in Figure 10.

Based on Figures 3, 4, 5, 6, 7, 8, 9 and 10, the tracking performances of the two kinds of receiver with different C/N0 are compared and listed in Table 2. The optimal PLL bandwidth of the ultra-tightly integrated GNSS/INS receiver is less than that of the standard receiver. The narrow bandwidth can reduce tracking phase noise, so the ultra-tightly integrated GNSS/INS receiver has better tracking performances than the standard receiver in GNSS-challenged environments. Furthermore, due to the reduction of the dynamic stress noise, the phase error of the PLL in the ultra-tightly integrated GNSS/INS receiver can decrease by 1–8 degree in different C/N0 scenarios. In this case, the PLLs of the ultra-tightly integrated GNSS/INS receiver are easier to be locked. Moreover, the dynamic stress noise is not considered as a crucial error source in the ultra-tightly integrated GNSS/INS receiver as its proportion decreases.

The biggest advantage of the ultra-tightly integrated GNSS/INS receiver is that it can perform in a highly dynamic scenario. Assumed that the C/N0 is 30 dB-Hz, Table 3 compares the tracking performances of two kinds of receiver with different dynamics based on Figures 5 and 6, Figures 8 and 9. In the standard receiver, the optimal bandwidth, the tracking error and dynamic stress noise proportion gradually increase as the receiver antenna's dynamics raise. But, the ultra-tightly integrated receiver has good tracking performances all the time and is not influenced by the receiver antenna's dynamics.

## 6. The Effect of the IMU Quality on the Tracking Performances

Based on the ultra-tight integration, the tracking performances of the receiver are enhanced by compensating the receiver antenna's dynamics and reducing the dynamic stress noise. The estimation accuracy of the dynamics is affected by the quality of the IMU. Therefore, the IMU quality becomes an impact factor in the tracking of the ultra-tightly integrated GNSS/INS receiver. The relation between the dynamic stress noise and the quality of the IMU, derived from Equations (12) and (13), is expressed for second-order PLL in the ultra-tightly integrated GNSS/INS receiver:

(15) $$\begin{array}{cc} \theta_{UT}=\frac{1}{3}\cdot K_{2}\frac{\bar{\nabla }+\bar{g}\cdot \bar{\alpha }\left(0\right)+\bar{g}\cdot \bar{ɛ}\cdot t_{d}}{{\left(B_{\text{PLL}}\right)}^{2}}\cdot \frac{360\cdot L_{c}}{c} & \left({}^{\circ}\right) \end{array}$$

The dynamic stress noises in four ultra-tightly integrated GNSS/INS receivers aided by different grade IMUs are compared to verify the effect of the IMU quality. Table 4 lists the IMUs selected.

**Figure 11.**
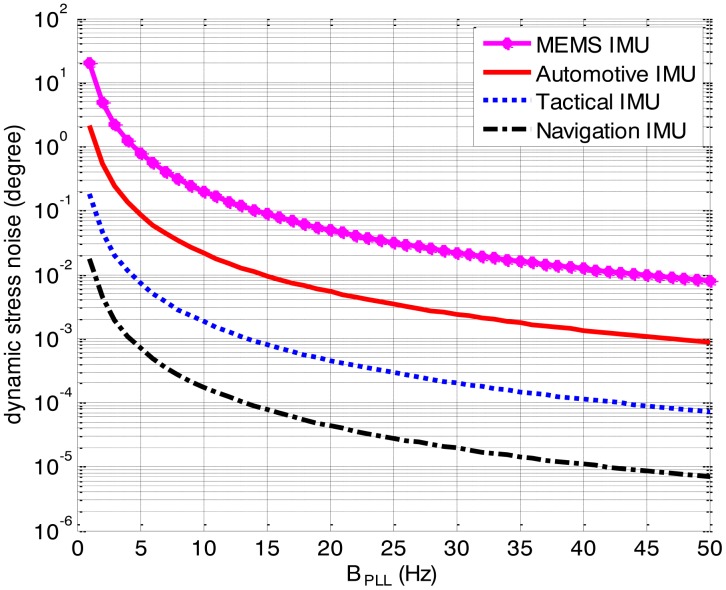
Dynamic stress noises in the ultra-tightly integrated GNSS/INS receivers aided by different grade IMUs.

Since the correction frequency of the INS in the ultra-tight integration is usually 1 Hz, the drift time *t_d_* is assumed to 1 s and initial misalignment is null. Figure 11 illustrates that the dynamic stress noise decreases as the IMU quality is enhanced. For a low quality IMU, such as the MEMS IMU and the automotive IMU, the IMU errors would have an effect on the loop noise and tracking performances of the PLL if a narrow bandwidth is selected in the ultra-tightly integrated GNSS/INS receiver. On the other hand, for a high quality IMU, such as the tactical IMU and the navigation IMU, the dynamic stress noise in the ultra-tightly integrated GNSS/INS receiver is far less than the tracking threshold and can be neglected. Therefore, to get better tracking performances, a high quality IMU should be used in the ultra-tightly integrated GNSS/INS receiver.

## 7. Conclusions

Based on the principle and definition of ultra-tight integration, this paper analyzes the performance improvements of the receiver tracking loops with Doppler aid from an INS. The models of every error source in the standard receiver and the ultra-tightly integrated GNSS/INS receiver are established and compared, respectively. By comparison of the tracking performances and the error distributions, we can conclude that the ultra-tight integration can improve the tracking performance of the receiver by compensating the receiver antenna's dynamics and reducing the dynamic stress noise. Moreover, the performance comparisons of the PLLs aided by different grade IMUs demonstrate that the quality of the IMU has an effect on the tracking performances in the ultra-tightly integrated GNSS/INS receiver.

## Figures and Tables

**Table 1.** Clock parameters of different oscillators.

**Oscillator**	*h*_0_[*s*]	*h*_−1_[−]	*h*_−2_[1/*s*]
TCXO	1.00 × 10^−21^	1.00 × 10^−20^	2.00 × 10^−20^
OCXO	2.51 × 10^−26^	2.51 × 10^−23^	2.51 × 10^−22^
Rubidium	1.00 × 10^−23^	1.00 × 10^−22^	1.30 × 10^−26^
Cesium	2.00 × 10^−20^	7.00 × 10^−23^	4.00 × 10^−29^

**Table 2.** Tracking performance comparisons of two kinds of receiver with different C/N0 (0.5 g LOS acceleration).

**C/N_0_**		**25 dB-Hz**	**30 dB-Hz**	**35 dB-Hz**	**40 dB-Hz**
Standard Receiver	Optimal Bandwidth	15 Hz	22 Hz	28 Hz	35 Hz
	Tracking Error of the PLL in Optimal Bandwidth	28 deg.	15 deg.	9 deg.	7 deg.
	Dynamic Stress Noise Proportion (B_PLL_ = 20 Hz)	8%	14%	21%	28%

Ultra-tightly Integrated GNSS/INS Receiver	Optimal Bandwidth	7 Hz	9 Hz	13 Hz	25 Hz
	Tracking Error of the PLL in Optimal Bandwidth	20 deg.	12 deg.	8 deg.	6 deg.
	Dynamic Stress Noise Proportion (B_PLL_ = 20 Hz)	<1%	<1%	<1%	<1%

**Table 3.** Tracking performance comparisons of two kinds of receiver with different dynamics (30 dB-Hz C/N0).

**Dynamics**		**0.1 g**	**0.5 g**	**1 g**	**1.5 g**
Standard Receiver	Optimal Bandwidth	14 Hz	22 Hz	28 Hz	32 Hz
	Tracking Error of the PLL in Optimal Bandwidth	13 deg.	15 deg.	17 deg.	18 deg.
	Dynamic Stress Noise Proportion (B_PLL_ = 20 Hz)	3%	14%	25%	33%

Ultra-tightly Integrated GNSS/INS Receiver	Optimal Bandwidth	9 Hz	9 Hz	9 Hz	9 Hz
	Tracking Error of the PLL in Optimal Bandwidth	12 deg.	12 deg.	12 deg.	12 deg.
	Dynamic Stress Noise Proportion (B_PLL_ = 20 Hz)	<1%	<1%	<1%	<1%

**Table 4.** IMUs selected in the comparisons.

	**MEMS IMU**	**Automotive IMU**	**Tactical IMU**	**Navigation IMU**
Gyro bias	300°/h	50°/h	1°/h	0.01°/h
Accelerometer bias	10 mg	1 mg	0.1 mg	0.01 mg

## References

[1] Gebre-Egziabher D. (2007). What is the difference between ‘loose’, ‘tight’, ‘ultra-tight’, and ‘deep’ integration strategies for INS and GNSS?. Inside GNSS.

[2] Groves P.D. (2008). Principles of GNSS, Inertial, and Multisensor Integrated Navigation Systems.

[3] Van Diggelen F., Abraham C. (2001). Indoor GPS Technology.

[4] Ohlmeyer E.J. Analysis of an Ultra-Tightly Coupled GPS/INS System in Jamming.

[5] Jwo D. (2001). GPS receiver performance enhancement via inertial velocity aiding. J. Navig..

[6] Gustafson D., Dowdle J., Flueckiger K. A Deeply Integrated Adaptive GPS-Based Navigator with Extended Range Code Tracking.

[7] Lewis D.E. Ultra-Tightly Coupled GPS/INS Tracking Performance.

[8] Lashley M., Bevly D.M. A Comparison of the Performance of a Non-Coherent Deeply Integrated Navigation Algorithm and a Tightly Coupled Navigation Algorithm.

[9] Groves P.D., Mather C.J., Macaulay A.A. Demonstration of Non-Coherent Deep INS/GPS Integration for Optimised Signal-to-Noise Performance.

[10] Lashley M., Bevly D.M. (2013). Performance comparison of deep integration and tight coupling. Navigation.

[11] Petovello M., O'Driscoll C., Lachapelle G. Ultra-Tight Integration of an IMU with GPS/GLONASS.

[12] Wang K., Li Y., Rizos C. The Feasibility of MEMS Inertial Sensors for Deep Integration of GPS and INS.

[13] Petovello M.G., Lachapelle G. Comparison of Vector-Based Software Receiver Implementations with Application to Ultra-Tight GPS/INS Integration.

[14] Petovello M.G., Sun D., Lachapelle G., Cannon M.E. Performance Analysis of an Ultra-Tightly Integrated GPS and Reduced IMU System.

[15] Lashley M., Bevly D.M., Hung J.Y. Analysis of Deeply Integrated and Tightly Coupled Architectures.

[16] Li T., Petovello M.G., Lachapelle G. Performance Evaluation of Ultra-Tight Integration of GPS/Vehicle Sensors for Land Vehicle Navigation.

[17] Abbott A.S., Lillo W.E. (2003). Global Positioning Systems and Inertial Measuring Unit Ultratight Coupling Method.

[18] Lashley M. (2009). Modeling and Performance Analysis of GPS Vector Tracking Algorithms. Ph.D. Thesis.

[19] Lashely M., Bevly D.M. Performance Comparision of Deep Integration and Tight Couping.

[20] Irsigler M., Eissfeller B. (2002). PLL tracking performance in the presence of oscillator phase noise. GPS Solut..

[21] Kaplan E.D. (1996). Understanding GPS Principles and Application.

[22] Jwo D. (2001). Optimization and sensitivity analysis of GPS receiver tracking loops in dynamics environments. IEE Proc. Radar Sonar Navig..

[23] Fu L., Chen Y. Performance and Stablity Analysis of INS/GPS Ultra-Tight Integration with INS Aided Receiver Tracking Loops.

[24] Filler R.L. The Acceleration Sensitivity of Quartz Crystal Oscillators: A Review.

[25] Silva P.F., Silva J.S., Caramagno A. IADIRA: Inertial Aided Deeply Integrated Receiver Architecture.

[26] Ye P., Zhan X., Fan C. (2011). Novel optimal bandwidth design in INS-assisted GNSS phase lock loop. IEICE Electron. Express.

[27] Kreye C., Eissfeller B., Ameres G. Architectures of GNSS/INS Integrations: Theoretical Approach and Practical Tests. http://forschung.unibw.de/papers/gawoe8szerl4vflebo15xjf338yybw.pdf.

